# Exploring miR-9 Involvement in *Ciona intestinalis* Neural Development Using Peptide Nucleic Acids

**DOI:** 10.3390/ijms21062001

**Published:** 2020-03-15

**Authors:** Silvia Mercurio, Silvia Cauteruccio, Raoul Manenti, Simona Candiani, Giorgio Scarì, Emanuela Licandro, Roberta Pennati

**Affiliations:** 1 Department of Environmental Science and Policy, Università degli Studi di Milano, 20133 Milano, Italy; Silvia.Mercurio@unimi.it (S.M.); raoul.manenti@unimi.it (R.M.); roberta.pennati@unimi.it (R.P.); 2 Department of Chemistry, Università degli Studi di Milano, 20133 Milano, Italy; emanuela.licandro@unimi.it; 3 Department of Earth Science, Environment and Life, Università degli Studi di Genova, 16132 Genova, Italy; 4 Department of Biosciences, Università degli Studi di Milano, 20133 Milano, Italy; giorgio.scari@unimi.it

**Keywords:** PNA, antisense therapy, invertebrate, nervous system development, tunicates, microRNA

## Abstract

The microRNAs are small RNAs that regulate gene expression at the post-transcriptional level and can be involved in the onset of neurodegenerative diseases and cancer. They are emerging as possible targets for antisense-based therapy, even though the in vivo stability of miRNA analogues is still questioned. We tested the ability of peptide nucleic acids, a novel class of nucleic acid mimics, to downregulate miR-9 in vivo in an invertebrate model organism, the ascidian *Ciona intestinalis,* by microinjection of antisense molecules in the eggs. It is known that miR-9 is a well-conserved microRNA in bilaterians and we found that it is expressed in epidermal sensory neurons of the tail in the larva of *C. intestinalis*. Larvae developed from injected eggs showed a reduced differentiation of tail neurons, confirming the possibility to use peptide nucleic acid PNA to downregulate miRNA in a whole organism. By identifying putative targets of miR-9, we discuss the role of this miRNA in the development of the peripheral nervous system of ascidians.

## 1. Introduction

The microRNAs (miRNAs) are small (19−25 nucleotides in length) non-coding RNAs, which have emerged as a fundamental class of potent regulatory molecules [1]. They are involved in many developmental and physiological processes [2], as well as in pathological conditions. Dysregulation of miRNAs is indeed associated with a variety of pathologies [3], and the possibility to regulate gene expression by interfering with miRNA networks is one of the most intriguing fields of novel drug development (miRNA-based therapeutics); miRNAs replacement or inhibition could represent an effective therapeutic approach in many diseases [4]. Although huge advances have been made in miRNA-based therapy, the in vivo stability of miRNA analogues is still one of the main challenges in this field [5,6].

In this regard, peptide nucleic acids (PNAs) are promising tools for interference with miRNA activities and restoration of normal expression of their targets [7,8]. PNAs are synthetic mimics of nucleic acids [9,10], in which the negatively charged sugar-phosphate backbone is replaced by the neutral *N*-(2-aminoethyl)glycine unit. A set of unique features, including high specific and selective binding affinity towards complementary DNA and RNA strands [11] and enzymatic resistance [12], makes PNAs particularly suitable for antisense therapy [13].

At present, data on the use of PNAs to target miRNAs are accumulating [14,15,16,17,18,19,20], demonstrating the potential of PNA for future therapeutic applications, as well as for studying miRNA functions. However, only a few studies have been performed in vivo. In mice, PNA-based inhibition of miR-155 was demonstrated to be highly efficient and sequence-specific. In the experimental animals, treatment with anti-miR-155 PNAs did not affect levels of unrelated miRNAs, and the effects of the PNA-based inhibition mainly recapitulated the effects of miR-155 genetic deletion [21]. More recently, our research group successfully downregulated miR-7 by injections of anti-miR-7 PNAs directly into the eggs of a basal chordate, the ascidian *Ciona intestinalis* [22].

Ascidians are marine filter-feeding animals closely related to vertebrates. They develop through a tadpole larva, which displays a typical chordate body plan [23], and are amenable model organisms for genetics, experimental embryology, and molecular biology [23,24]. The genome of *C. intestinalis*, the most studied ascidian, retains the original form that existed before vertebrate genome duplications [25], keeping all the major vertebrate miRNA families [26]. Among these, miR-9 is a well-conserved miRNA that plays essential roles in developing and adult nervous system [27]. In *Ciona robusta*, previously known as *C. intestinalis* type A [28], miR-9 was found to be expressed at gastrula and larval stages [26]. In this species, through a transcriptome-wide study of gene expression of both mRNA and microRNA, it was found that miR-9 is upregulated during oral siphon regeneration and is involved in the differentiation of neural progenitors through regulation of the cytoskeleton and cell cycle [29]. This pro-neural function of miR-9 is conserved between ascidians and vertebrates, in which one of its key functions is to regulate the generation of postmitotic neurons from neural precursor cells [30]. In other invertebrate models, such as *Drosophila melanogaster*, miR-9 expression and functions are strikingly different from vertebrates, suggesting that miR-9 roles have undergone vast changes during evolution [27,31,32].

In humans, miR-9 is also involved in neurodegenerative diseases and cancers. Deregulation of miR-9 levels was observed in different neural pathologies; high levels of miR-9 were found in postmortem brains of patients with Alzheimer’s diseases [33], whereas miR-9 is downregulated in Huntington’s diseases [34]. Altered expression of miR-9 affects brain tumors, as well as breast and colorectal cancer formation and progression [35], making this miRNA a perfect candidate in cancer therapy [36].

Thus, considering the therapeutic potential of miR-9 and the advantages of PNA-based approaches, in this study we aim to investigate the ability of a PNA specifically designed to target miR-9 to inhibit its function in vivo, using *C. intestinalis* as a model organism [22]. Moreover, we evaluated the effects of this inhibition on the development of the *C. intestinalis* nervous system and proposed a possible mechanism of function by identifying its putative targets. This work adds precious information about conserved functions of miR-9 in chordates.

## 2. Results

### 2.1. miR-9 and PNAs

**Figure 1:**
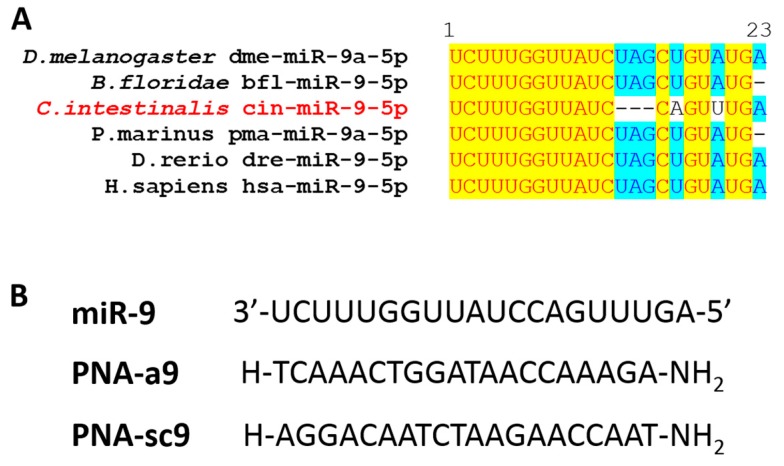
(**A**) Multialignment of miR-9 from different species. Identical residues are highlighted in yellow, and those conserved in at least 50% of sequences in light blue. The mature miR-9 sequences were retrieved from miRBase: dme-miR-9a-5p: MIMAT0000114; bfl-miR-9-5p: MIMAT0009465; cin-miR-9-5p: MIMAT0016396; pma-miR-9a-5p: MIMAT0019373; dre-miR-9-5p: MIMAT0001769; hsa-miR-9-5p: MIMAT0000441. (**B**) PNA sequences used in this study: PNA-a9 and PNA-sc9.

The miR-9 sequence is highly conserved among metazoans. *Ciona intestinalis* mature miR-9 is 20 bases long; the first 15 bases are perfectly conserved among different chordate species, as well as the last three nucleotides at the 3′ end. Therefore, at the 5′ region, the seed region appears to be evolutionarily conserved (Figure 1A).

Anti-miRNA PNAs are designed to be a perfect reverse complement to the mature miRNAs. In this study, we designed a 20-mer PNA that is complementary to miR-9—PNA-a9 (Figure 1B). Moreover, to verify the specificity of the interaction between PNA-a9 and miR-9, a scrambled anti-miR-9 PNA, PNA-sc9, was designed, which shares the same base composition with the specific sequence, however exhibited a randomized sequence (Figure 1B). PNA-a9 and PNA-sc9 were synthesized according to standard protocols for solid-phase synthesis of PNA, and the final sequences were purified by reverse phase-high pressure liquid chromatography (RP-HPLC).

### 2.2. miR-9 Expression during C. intestinalis Development

**Figure 2:**
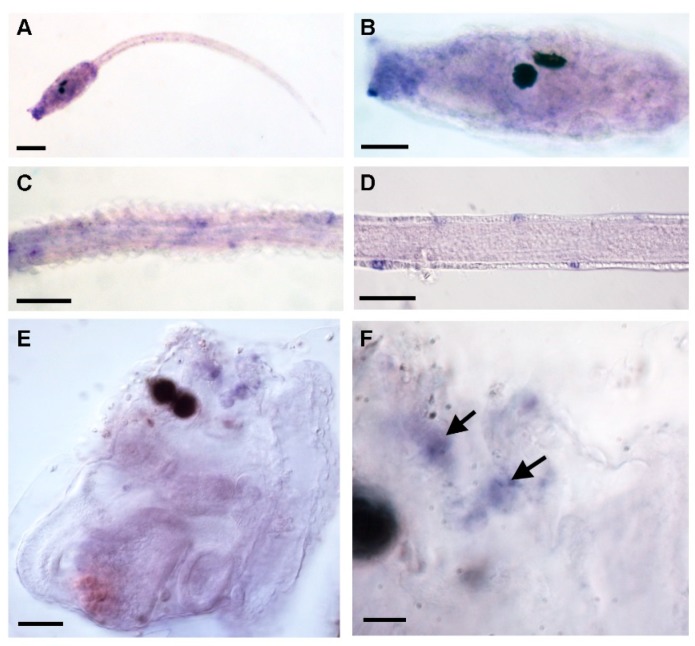
Whole mount in situ hybridization with pri-miR-9 (**A**–**C**) and anti-miR-9 locked nucleic acid -LNA probe (**D**–**F**) in larvae and juveniles of *Ciona intestinalis*. (**A**) Swimming larva. (**B**) Trunk of a larva. (**C**,**D**) Details of the tail of a swimming larva. (**E**) Whole mount of 5-day-old juvenile. (**F**) Details of the oral siphon region of a juvenile. Arrows indicate positive cells. Scale bar = 50 µm (**A**), 25 µm (**B**), 40 µm (**C**,**D**), 25 µm (**E**), 60 µm (**F**).

In order to determine miR-9 expression in developing C. intestinalis embryos, we performed whole mount in situ hybridization with a specific locked nucleic acid (LNA) probe for mature Cin-miR-9 (accession: MIMAT0016396). In swimming larvae, the hybridization signal was localized in the epidermal sensory neurons of dorsal and ventral midlines of the tail (Figure 2D). These results were confirmed by in situ hybridization with a probe specific for pri-miR-9 (Figure 2A–C). In juveniles, the miR-9 signal was detectable in several cells scattered in the epidermis near the oral syphon (Figure 2E,F).

### 2.3. miR-9 Downregulation by PNAs

To investigate the role of miR-9 in development, microinjections of PNA-a9 and PNA-sc9 (used as controls) were performed in *C. intestinalis* eggs. No difference in the developing rates was observed between embryos microinjected with PNA-a9 (65%) and controls injected with the medium (72%) and with PNA-sc9 (66%). Most of the embryos injected with PNA-a9 displayed a rounded and shorter trunk than controls (70%). The morphology of the embryos injected with PNA-sc9 was not affected by the treatment (78%), being comparable with the controls injected with the medium.

**Figure 3:**
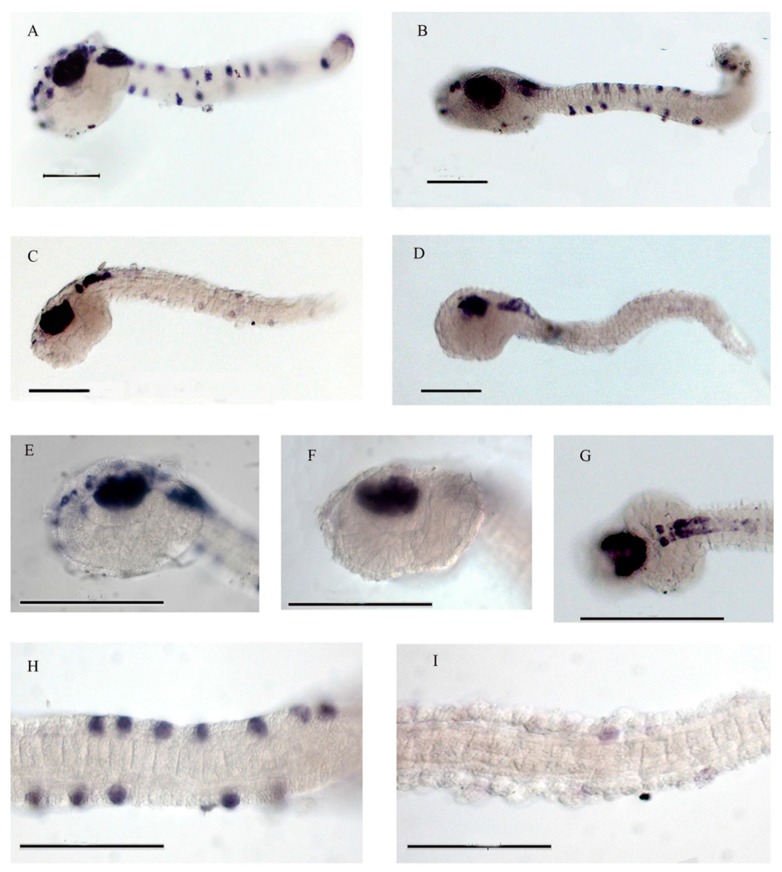
Whole-mount in situ hybridization with *Ci-Pans* probe. (**A**) Control and (**B**) PNA-sc9-injected embryos reveal similar expression. (**C**,**D**) Two lateral views of PNA-a9-injected embryos displaying a marked decrease of the signal in the epidermal sensory neurons. At higher magnification, the decrease of the signal in peripheral neurons induced by PNA-a9 is particularly evident in the trunk ((**E**) control; (**F**,**G**) injected samples) and tail ((**H**) control; (**I**) injected embryo). Scale bar, 100 µm for all panels.

When we analyzed the expression of neural marker genes, PNA-a9-injected embryos showed anomalies at the level of the peripheral nervous system. Indeed, in situ hybridization with a pan neural marker, *Ci-Pans* [37], revealed a reduced expression in the epidermal sensory neurons of both the trunk and tail (80%; Figure 3). Particularly, in the tail, transcripts were absent in the proximal dorsal and ventral epidermal neurons, however some neurons were eventually marked in the ventral tip of the tail (Figure 3C,D,F,G,I). Similarly, anterior sensory neurons of the trunk, including the papillary sensory neurons, were reduced in PNA-a9-injected embryos (Figure 3D,F).

**Figure 4:**
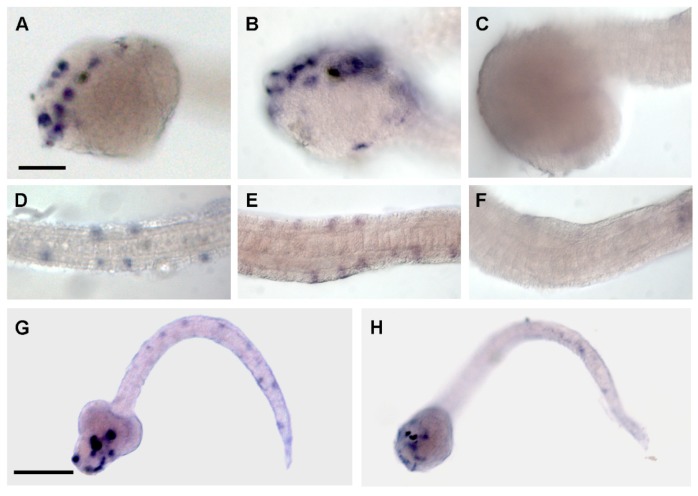
Whole mount in situ hybridization of control larvae (**A**,**B**,**D**,**E**,**G**) and larvae developed from embryos injected with PNA-a9 (**C**,**F**,**H**). (**A**–**F**) Anti-*Ci-POU IV*. (**G**–**H**) Anti-*Ci-V-Glut* probe.

We further characterized the effects of PNA-a9 injection; samples were processed for in situ hybridization with *Ci-POU-IV* and *Ci-V-Glut* probes. *Ci-POU-IV* is a gene involved in neural precursor specification and is a marker of the epidermal sensory neurons [38] (Figure 4A,B,D,E). *Ci-POU-IV* expression was completely absent in the tail of 80% of the larvae developed from injected eggs with PNA-a9 and only a few isolated neurons were faintly marked in the ventral midline (Figure 4C,F), confirming previous results. *Ci-V-Glut* [39] marks the sensory neurons of the papillae and the tail, the posterior visceral ganglion, and the posterior neural tube in the controls (Figure 4G). In injected larvae, the expression of this gene was evident only in a few neurons of the proximal neural tube, very close to the trunk (90% of tested embryos; Figure 4H)

### 2.4. miR-9 in Silico Targets Identification

In order to understand the role of miR-9 in epidermal sensory neuron (ESN) differentiation, we searched for putative targets among genes known to be involved in this process in *C. intestinalis* [40] and in common pathways of vertebrates [27]. We identified as putative targets of miR-9: (a) *Ci-Hes-b* (AK174862), *Ci-SoxB1* (NM_001128858), and *Ci-SoxB2* (NM_001128857), as they are known to regulate ESN differentiation in *Ciona* [40]; (b) *Ci-FoxG* (XM_002124957), since its homolog, *FoxG1*, has been demonstrated to be a specific neural target of miR-9 [41]; (c) *Ci-Cadherin* (XM_009861227) and *Ci-Cadherin 7* (XM_026837209), as miR-9 is the only known miRNA to target *E-cadherin* transcript in vertebrates [35].

Then, we tested these selected transcripts by bioinformatics analysis. At present, accurate in silico identification of miRNA targets is a great challenge. Numerous programs have been developed to predict putative miRNA targets in vertebrates, *Drosophila*, and worms (e.g., TargetScan, DIANA, miRANDA, etc.), whereas only a few can be employed in non-standard animal models. In this study, we used 3 free available programs: RNAhybrid [42], RNA22 version 2.0 [43], and PITA executable [44].

We found one or more target sites in all analyzed transcripts. Only considering sites within 3′UTR, all 3 programs identified the same target site in *Ci-FoxG*, whereas 2 programs predicted the same target site in *Ci-Hes-b*, *Ci-caderin*, and *Ci-SoxB1/2* mRNAs (Table 1).

## 3. Discussion

We explored miR-9 involvement in the *C. intestinalis* development, performing knockdown experiments with antisense peptide nucleic acids (PNAs). PNAs are nucleic acid analogues characterized by a synthetic peptide backbone. These oligomers specifically bind to complementary DNA or RNA sequences, obeying Watson–Crick base paring [11]. In recent years, different studies have demonstrated PNA specificity for their complementary miRNAs in vitro [4,7,8].

In ascidians, recent comparisons between PNAs and the commercial AntagomiRs (Dharmacon, USA) antisense molecules have demonstrated the ability of PNAs to efficiently downregulate miRNAs. The effects induced by injections of these molecules (PNAs and AntagomiRs) into *C. intestinalis* eggs were comparable, confirming the reliability of PNA-based knockdown [22].

Modified PNAs were also used for sequence-based knockdown mRNA translation through the same morpholino phosphorodiamidate (MO) mechanism of action in zebrafish. Studies comparing the strength of these molecules demonstrated that they were equally effective in downregulating target expression. In addition, PNA oligomers proved to be more specific than conventional MO: even a one-base mismatch in PNA was sufficient to prevent PNA binding with target sequences, while three mismatches were necessary to prevent MO inhibitory effects [45].

Furthermore, miR-9 is one of the most conserved miRNAs in bilaterian animals, characterized by specific expression in the nervous systems of both vertebrates and invertebrates [27,46,47]. According to our analyses, in the developing nervous system of the ascidian *C. intestinalis*, mature miR-9 is present in epidermal sensory neurons of the tail and in the papillary sensory neurons. This expression was confirmed using a probe for pri-miRNA. It could be possible that miR-9 is also expressed at low levels in other regions of the developing embryos and cannot be detected by in situ hybridization. We found that downregulation of miR-9 by PNA-a9 microinjections in eggs specifically affected the development of the peripheral nervous system (PNS), as demonstrated by in situ hybridization with specific markers. Although quantitative analysis would be necessary to completely confirm PNA-a9 efficiency, the results appear to be highly reliable. Moreover, we never observed the expression of *Ci-POU IV*, which is required for PNS specification, in the epidermis of the tail and the trunk of injected embryos.

The PNS of ascidian larvae is composed of a limited number of epidermal sensory neurons (ESNs) located in the trunk and in the tail midline. These ciliated neurons are thought to have mechanosensory functions. In the tail, dorsal and ventral ESNs arise from two separate populations of cells with partially different gene circuits [40,48,49]. The tail epidermis midline is a neurogenic region and the Notch pathway negatively controls the number of cells adopting the ESN fate [48]. Subsequently, the sequential activation of MyT1, POU IV, atonal, and NeuroD induces ESN development. Larvae injected with PNA-a9 showed few or no differentiated ESNs. It could be hypothesized that downregulation of miR-9 repressed neural fate determination, as it inhibited neural differentiation by ectopic expression of some of its targets.

Two inhibitory mechanisms have been proposed for preventing ESN formation; one involving Notch effector genes, such as those belonging to Hes transcriptional repressor family, and the other through *SoxB2* gene activation [40]. Our bioinformatics analysis indicated that *Hes* is a putative target of miR-9 in *C. intestinalis*, differing from the results of Spina et al. (2011), who did not identify binding sites for miR-9 in any members of the Notch pathway. Interestingly, a set of miR-9 targets are members of the *Hes* gene family in vertebrates, where they inhibit neural differentiation by repressing proneural genes [27]. The *C. intestinalis* genome contains three *Hes* genes (*Hes-a*, *Hes-b*, *Hes-c*) with different patterns of expression [40,50]. Particularly, *Hes-b* is expressed outside the epidermis midlines, probably preventing ESN formation [40]. Downregulation of miR-9 could induce *Hes-b* ectopic expression in the midline, thus inhibiting PNS differentiation in this region. *SoxB2* is also a putative target identified by our analysis, and it is expressed throughout the epidermis but not in the midlines. Although few studies are available about this gene in ascidians, it has been demonstrated that *MsxB* knockdown caused *SoxB2* upregulation in the dorsal midline [51]. Moreover, in *C. intestinalis*, *SoxB2* transcripts have been found to be a target of miR-124, another essential neural miRNA, suggesting that its downregulation is necessary to allow nervous system differentiation [40,52].

It is known that miR-9 is the only predicted miRNA to target *E-cadherin* transcripts in vertebrates [35]. This transmembrane glycoprotein forms the core of cellular adherens junctions and sequesters β-catenin from the cytoplasm. In *C. intestinalis*, cadherin superfamily genes have been characterized and include 2 classical cadherins: *Cadherin* and *Cadherin II* [53]. Our in silico investigations revealed that *Cadherin*, but not *Cadherin II*, could also be a possible target of miR-9 in ascidians. In addition, it has been reported that *Cadherin* overexpression decreases the cytoplasmatic and nuclear levels of β-catenin, a transcription factor involved in endoderm specification. Disturbance in endoderm differentiation has been demonstrated to lead to differentiation of excess epidermis cells [54]. Thus, miR-9 could also act through Wnt pathways, participating in cell fate determination during ESN development.

Both bioinformatics programs used in this study predicted the same miR-9 target site in the *FoxG* (the *forkhead transcription factor*) transcript. Among tetrapods, the miR-9 target sequence at *FoxG1* 3′UTR (previously called BF-1) is highly conserved [41]. This gene is present in the *C. intestinalis* genome, however no data are available about *FoxG* expression and functions. In vertebrates, it is expressed in the telencephalic region of the neural plate and its expression persists in the adult telencephalon. During embryonic development, it has been shown to be essential in driving ventral telencephalic fate downstream of Hh/Fgf pathways and controlling the extent of the dorsal compartment through direct transcriptional repression of Wnt ligands [55]. In the cephalochordate, amphioxus *AmphiBF-1* expression has been characterized, and notably, at 3 days of development, it is expressed in the anterior-ventral part of the cerebral vesicle [56]. Conservation of the *FoxG1* expression pattern in cephalochordates and vertebrates suggests that this gene and its regulatory network could be evolutionarily conserved. However, the *C. intestinalis FoxG* target sequence shows only partial similarities with vertebrates, and further specific analyses are needed to solve this hypothesis.

Overall, our results showed miR-9 involvement in ascidian neural development, further underlining the evolutionary conservation of both miRNA sequences and miRNA-mediated post-transcriptional pathways between vertebrates and ascidians, which have already been suggested by other authors [40,52]. In addition, we described in vivo biological activity of a PNA oligomer directed against the miRNA-9 in *C. intestinalis* embryos. Our results suggest that anti-miR PNA-a9 is able to reach its specific target in the developing ascidian embryos with high efficiency, as underlined by the lack of effect induced by the scrambled sequence. This is further evidence that an unmodified PNA can be successfully used in knockdown strategies in the multicellular organisms of ascidian larvae.

## 4. Materials and Methods

### 4.1. Synthesis and Characterization of PNAs

PNA-a9 and PNA-sc9 were synthesized by manual solid-phase synthesis using Boc/Z chemistry [57]. The commercially available Boc/Z-protected PNA monomers were purchased from ASM Research Chemicals GmbH (Hannover, Germany). The MBHA resin was purchased from VWR International (Milan, Italy), and it was loaded manually to 0.2 mmol/g with Boc/Z-adenine PNA monomer for PNA-a9 and with Boc-thymine PNA monomer for PNA-sc9, following the previous procedure [58]. The PNA purification was performed by reverse-phase RP-HPLC with an Agilent 1200 Series system, equipped with a diode array detector (UV detection at 260 nm). The purity of PNA-a9 and PNA-sc9 was checked by RP-HPLC analyses, and their identities were confirmed by MALDI-TOF mass spectra, which were recorded with a Bruker Daltonics Omniflex (Milan, Italy), equipped with a 337 nm pulsed nitrogen laser (sinapinic acid as matrix). PNA-a9 calculated MW for C_216_H_266_N_124_O_53_: 5447.2; MALDI-TOF MS found *m*/*z* 5448.6 [M+H]^+^. PNA-sc9 calculated MW for C_216_H_266_N_124_O_53_: 5447.2; MALDI-TOF MS found *m*/*z* 5448.4 [M+H]^+^.

### 4.2. Animals and Embryos Microinjections

*Ciona intestinalis* adults were collected along the coasts of Roscoff by the fishing service of the Roscoff Biological Station (Roscoff, France). Animals were maintained in aquaria filled with artificial sea water (Instant Ocean; salinity about 32‰) and provided with a circulation system, as well as mechanical, chemical, and biological filters. Constant light condition was preferred to promote gamete production. Gametes from three adults were obtained surgically from the gonoducts and reared in artificial sea water buffered with 1M HEPES (ASWH; pH 8.0) at 18 ± 1 °C until the stages of interest. Before being fixed in 4% paraformaldehyde, 0.5 M NaCl, and 0.1 M 3-(N-morpholino)propanesulfonic acid, embryos at gastrula, neurula, and tailbud stages were dechorionated in ASW containing 1% sodium thioglycolate and 0.05% protease.

For PNA microinjections, only batches in which 90% or more of the eggs developed normally were used. Dechorionated eggs were microinjected with solutions of 0.7 mM and 1 mM PNAs (PNA-a9 and PNA-sc9) in distilled water, plus 5 μg/μL Fast Green as the vital dye, as described previously [59]. In vitro cross-fertilization was performed and embryos were reared at 16 °C in a 1% agarose-coated petri dish in ASW until they reached late tailbud and larva stages (16 and 22 h post fertilization) [60].

### 4.3. In Situ Hybridization

To describe Cin-miR-9 (MIMAT0016396) expression during *C. intestinalis* development, in situ hybridization with a DIG-labeled locked nucleic acid (LNA; Exiqon, Vedbaek, Denmark) probe (cin-miR-9: 5′-TCAAACTGGATAACCAAAGA/3Dig_N; DNA Tm = 74 °C) was carried out as described before [22].

To confirm the result, a DIG riboprobe specific for pri-miR-9 was also synthetized. First, a PCR product of 578 bp was amplified from *C. intestinalis* genomic DNA using 5′-TACTGGAGCGTGTTAGGTTTATTG-3′ and 5′-AGGGATGCCATGATTAGTAGTGAC-3′ as forward and reverse primers, respectively. Then, a fragment of 357 bp was obtained by performing a nested PCR with 5′-AACAACGGCCGTATTGCTTT-3′ (forward) and 5′-ACCCCAAATGCTGTTTCGTG-3′ (reverse). After cloning with TOPO TA Cloning Kit (Thermo Fisher Scientific, Milan, Italy), DIG-labelled antisense and sense riboprobes were transcribed with Sp6 and T7 RNA polymerase, using a DIG RNA labelling kit (Roche, Monza, Italy).

Standard in situ hybridizations with riboprobes were performed [61]. To characterize the effects of PNA injections, the following neural marker genes were employed: *Ci-Pans* [37], *Ci-POU IV* [38], and *Ci-V-Glut* [39]. For each probe, at least 25 injected and control embryos were analyzed. For each experimental group, the percentages of samples with abnormal expression were calculated as (number of samples with altered expression / total number of analyzed samples) × 100.

### 4.4. miR-9 Target Prediction

Based on literature [27,40], we identified *Ci-Hes-b* (AK174862), *Ci-SoxB1* (NM_001128858), *Ci-SoxB2* (NM_001128857), *Ci-FoxG* (XM_002124957), *Ci-Cadherin* (XM_009861227), and *Ci-Cadherin 7* (XM_026837209) as potential neural targets of miR-9. We used three target prediction programs, namely RNAhybrid [42], RNA22 version 2.0 [43], and PITA executable [44], in order to increase the accuracy of target identification. Bioinformatic algorithms were run with default parameters. Transcripts with a binding site in the 3′-UTR region found by one of the bioinformatic algorithms were considered putative miR-9 targets.

## Figures and Tables

**Table 1:** List of the results obtained from in silico analysis of miR-9 putative targets in *C. intestinalis*. RNAhybrid, RNA22, and PITA programs. A target was considered valid when found inside the 3′UTR region.

	3′UTR	RNAhybrid	RNA22	PITA	Target Position
*Ci-Hes-B*	1101…1433	X	-	X	1192
*Ci-SoxB1*	1392…2584	X	-	X	1468
*Ci-SoxB2*	1136…2983	X	-	X	2010
*Ci-FoxG*	1742…1964	X	X	X	1835
*Ci-Cadherin*	2544…3215	X -	X -	X X	2558 2791 *
*Ci-Cadherin 7*	2837…3160	X	-	-	3085

* target site predicted only by PITA.

## Abbreviations

Boc	*tert*-Butyloxycarbonyl
MALDI-TOF	Matrix-assisted laser desorption/ionization time-of-flight
MBHA	4-Methylbenzhydrylamine hydrochloride
RP-HPLC	Reverse-phase high-pressure liquid chromatography
Z	Benzyloxycarbonyl
